# A Novel Acetylene-Functional/Silicon-Containing Benzoxazine Resin: Preparation, Curing Kinetics and Thermal Properties

**DOI:** 10.3390/polym12050999

**Published:** 2020-04-26

**Authors:** Qilin Mei, Honghua Wang, Danliao Tong, Jiuqiang Song, Zhixiong Huang

**Affiliations:** School of Materials Science and Engineering, Wuhan University of Technology, 122 Luoshi Road, Wuhan 430070, China; meiqilin@whut.edu.cn (Q.M.); 13343434177@163.com (H.W.); 127565@whut.edu.com (D.T.); Songjq9@126.com (J.S.)

**Keywords:** benzoxazine, acetylene, silicon, curing kinetics, thermal stability

## Abstract

Benzoxazine resin has been paid more attention in the fields of aviation, electronics, automobiles and new energy industries because of its excellent comprehensive performance. Further application is limited, however, by shortcomings such as high brittleness and high curing temperature. Furthermore, higher thermal stability is imperiously demanded in special areas. Incorporating both an acetylene group and silicon into the benzoxazine monomer is a promising possible solution to improve the curing processability, thermal properties and toughness of benzoxazine. In this paper, an acetylene-functional/silicon-containing benzoxazine monomer was prepared by two-step synthesis, and acetylene-functional benzoxazine was also prepared as a comparison. FTIR and ^1^H NMR confirmed the molecular structure of acetylene-functional/silicon-containing benzoxazine. Differential scanning calorimetry (DSC) analysis showed that the initial and peak degradation temperatures of acetylene-functional/silicon-containing benzoxazine were decreased by 21 °C and 18 °C compared with acetylene-functional benzoxazine, respectively. The apparent activation energy of the curing reaction of acetylene-functional/silicon-containing benzoxazine was 83.1 kJ/mol, which was slightly lower than acetylene-functional benzoxazine (84.7 kJ/mol). TGA results showed that the acetylene-functional/silicon-containing benzoxazine had a higher thermal stability than acetylene-functional benzoxazine. The temperatures of 5% weight loss of acetylene-functional/silicon-containing benzoxazine were 380 °C in nitrogen and 485 °C in air, and the char yield at 1000 °C was 80% in nitrogen and 21% in air, respectively. The results of mechanical properties showed that the impact strength of acetylene-functional/silicon-containing benzoxazine was higher than acetylene-functional benzoxazine by 35.4%. The tensile and flexural strengths of acetylene-functional/silicon-containing benzoxazine were slightly higher than that of acetylene-functional benzoxazine.

## 1. Introduction

Fiber-reinforced polymer composites with high thermal stability have been widely applied in aerospace, electronics, automobiles and new energy industries because of their low density, high temperature resistance, high strength and high modulus [1,2,3]. The thermal stability of fiber-reinforced polymer composites is mainly determined by the matrix resin. The matrix resin must maintain a reliable structure and provide good mechanical properties at high temperatures to avoid defects, such as deformation, cracking and even degradation. As a novel high-performance thermosetting phenolic resin, benzoxazine not only retains the advantages of general phenolic resin, including heat stability, good mechanical properties, and electrical properties [4], but also possesses the properties of molecular design flexibility, not releasing any byproduct in the curing process, low curing shrinkage rate, low water absorption and high char yield. These excellent comprehensive properties widen its application in the aviation, electronics, automobile and new energy industries [5,6].

Despite these advantages, benzoxazine has a few defects, such as high brittleness and poor processability [7,8,9]. It is noteworthy that benzoxazine has a new oxazine ring, which traditional phenolic resin does not possess, resulting in increasing density of the cross-linked network. Nevertheless, it is still a brittle material compared with other common high-performance materials, such as epoxy resin, polyimide, polyphenyl ether etc. Moreover, the thermal stability of benzoxazine is not enough to meet the high requirements for materials in aerospace and power electronics. As a result, it is necessary to further enhance the thermal stability to widen its application. Also, the curing temperature of benzoxazine is obviously higher and the curing time is longer than traditional phenolic resins. The low production efficiency and high production cost are the obstacles for its widespread application. Therefore, decreasing the curing temperature is the main topic of research in the sustainable development of benzoxazine.

As benzoxazine has a good molecular design flexibility, some reactive functional groups and flame retardant elements have been introduced into the molecular structure to improve the exclusive property [10,11,12,13,14]. Hatsuo I. et al. [15,16] synthesized a novel acetylene-functional benzoxazine resin, of which the high char yield achieved was in the range of 71%–81% by weight at 800 °C in a nitrogen atmosphere, which was mainly contributed by the acetylene polymerization. Zhang J. et al. [17] prepared a copolymer of silicon-containing arylacetylene resin and acetylene-functional benzoxazine, of which the cross-linking reactions were due to the (1) Diels–Alder reaction between Ph-C≡C and C≡C, (2) ring trimerization of C≡C and (3) radical polymerization of C≡C to form a polyene structure. Xu Y. et al. [18] prepared a polybenzoxazine modified by acetylene and aldehyde groups, and its glass transition temperature (T_g_) from dynamic thermomechanical analysis (DMA) and char yield (Y_c_) at 800 °C in a nitrogen atmosphere were as high as 459 °C and 77.2%, respectively. Also, the presence of the aldehyde group led to a low curing temperature and wide processing window, which were beneficial to processing.

The silicon-containing benzoxazine possesses good thermal stability and has been greatly applied in ceramic precursors, heat-resistant materials and the matrix of high-performance polymer composites in aerospace and astronautics [19,20,21,22]. Gao Y. et al. [23] prepared blends of silicon-containing arylacetylene resin and benzoxazine by physical mixture, and the cured blends exhibited high T_g_ and thermal stabilities. Also, the compressive strength and flexural strength of the blend were improved. Zeng K. [24] synthesized a silicon-containing benzoxazine, and the results showed that the silicon-containing polybenzoxazine possessed a significantly higher initial degradation temperature and char yield than the conventional bisphenol A/aniline-based polybenzoxazine.

In order to improve the overall curing processability, thermal properties and toughness of benzoxazine, incorporating both an acetylene group and silicon into the benzoxazine monomer is a promising possible solution. In this paper, an acetylene-functional/silicon-containing benzoxazine monomer was prepared by two-step synthesis. The molecular chemical structures were investigated by Fourier transform infrared (FTIR) spectroscopy and proton nuclear magnetic resonance (^1^H NMR) spectra. The viscosity–temperature relationship was measured by a TA instrument. The curing kinetics were studied by differential scanning calorimetry (DSC). The thermal stability was researched by using a thermogravimetric analyzer (TGA) to identify the effects of acetylene and silicon on the thermal stability of benzoxazine. The mechanical properties were tested to identify the influence of the ester group on toughness.

## 2. Experimental Section

### 2.1. Materials

Hydroquinone (AR, purity 99%), pyridine (AR, purity 99.5%), 1,4-dioxane (AR, purity 99.5%), methylbenzene (AR, purity 99%), tetrahydrofuran (AR, purity 99%) and formaldehyde (AR, purity 99.5%) were purchased from Sinopharm Chemical Reagent Co. Ltd. (Shanghai, China). Dimethyldichlorosilance (GC, purity 98%) and M-aminophenyl acetylene (purity 98%) were provided by Aladdin Industrial Co. Ltd. (Shanghai, China). All the chemicals were used as received without further purification.

### 2.2. Synthesis of Phenolic Hydroxyl-Terminated Siloxane (PHS)

**Scheme 1:**
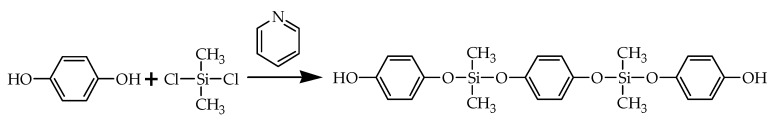
Synthesis of phenolic hydroxyl-terminated siloxane (PHS).

The synthesis process of PHS is shown in Scheme 1. Hydroquinone (20.02 g, 0.2 mol) and pyridine (24 g, 0.3 mol) were dissolved in methylbenzene (40 mL). The solution was charged into a 500 mL round-bottom flask. Then, the dimethyldichlorosilance (19.35 g, 0.15 mol) was dropwise added into the flask. The reaction was maintained at 80 °C for 5 h in a nitrogen atmosphere. After that, 100 mL distilled water was added into the flask to dissolve the product. The aqueous phase was removed by a 500 mL separatory funnel, and a white viscous liquid was obtained. The white viscous liquid was dried under a vacuum to afford a light-yellow solid. The light-yellow solid was dissolved in tetrahydrofuran and precipitated by distilled water several times to obtain pure phenolic hydroxyl-terminated siloxane (PHS) with a yield of about 85%. The number average molecular weight (Mn) of PHS is 684 g/mol, with a polydispersity distribution index of 1.7.

### 2.3. Synthesis of Acetylene-Functional/Silicon-Containing Benzoxazine Resin (ASB)

**Scheme 2:**
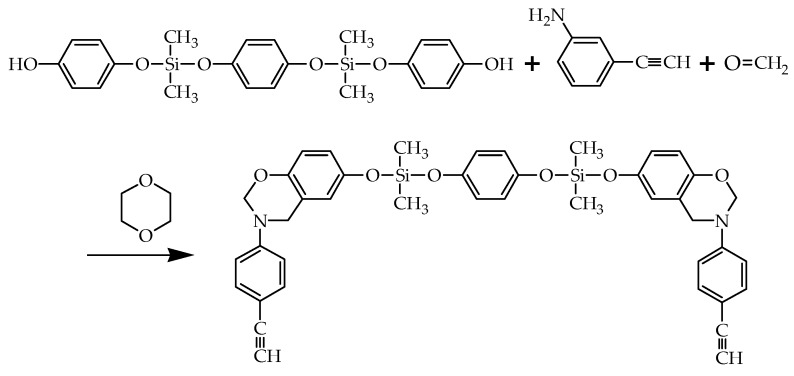
Synthesis of acetylene-functional/silicon-containing benzoxazine resin (ASB).

The whole reaction process of the synthesis of ASB was protected by a nitrogen atmosphere, and the synthesis route is shown in Scheme 2. Formaldehyde (1.20 g, 0.04 mol) and 1,4-dioxane (20 mL) were added into a 500 mL round-bottom flask. Then, a solution of M-aminophenyl acetylene (1.18 g, 0.01 mol) dissolved in 1,4-dioxane (20 mL) was dropwise added into the flask in 30 min under ice bath conditions. After that, a solution of PHS (4.42 g, 0.01 mol) dissolved in 1,4-dioxane (20 mL) was added into the flask and reacted at 90 °C for 6 h. The residual was collected and dried under a vacuum at 40 °C to obtain the solid product. The solid product was dissolved in methylbenzene and initially washed with sodium hydroxide solution and then finally washed with distilled water. Rotary evaporator and vacuum drying, respectively, were utilized to get rid of methylbenzene and water to obtain the black solid of ASB with a yield of about 75%. The Mn of ASB is 843 g/mol, with a polydispersity distribution index of 2.2. ^1^H NMR (400 MHz, CDCl_3_, d): 0.14 (m, 12H, Si-CH_3_), 3.06 (s, H, C≡CH), 4.64 (m, 4H, Ar-CH_2_-N), 6.00 (m, 4H, N-CH_2_-O). 

**Scheme 3:**
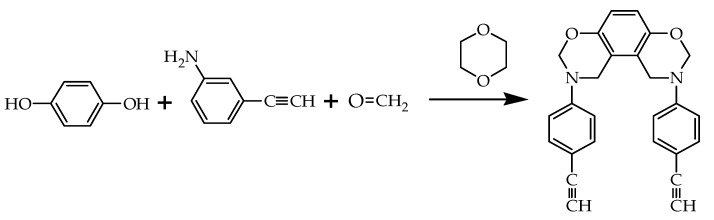
Synthesis of acetylene-functional benzoxazine resin (AB).

The synthetic process of acetylene-functional benzoxazine resin (AB) with a yield of about 82% is similar to that of ASB as shown in Scheme 3. The Mn of AB is 387 g/mol, with a polydispersity distribution index of 1.2. 

### 2.4. Preparation of Polybenzoxazines 

The mold was preheated in an oven at 150 °C for 0.5 h. The release agent was evenly sprayed on the surface of mold. Then, ASB (230 g) and AB (230 g) were put into the mold. The ASB was cured in an air-circulating oven by the following steps: 150 °C (2 h), 170 °C (2 h), 190 °C (2 h), 210 °C (2 h), and post-curing at 260 °C (3 h). The AB was cured in an air-circulating oven by the following steps: 150 °C (2 h), 170 °C (2 h), 190 °C (2 h), 215 °C (2 h), 240 °C (2 h) and post-curing at 260 °C (3 h). 

### 2.5. Instruments

Fourier transform infrared (FTIR) spectroscopy was recorded on KBr pellets from 4000 to 400 cm^−1^ by a Nicolet Nexus IR Spectrometer, made in Madison, WI, USA. Proton nuclear magnetic resonance (^1^H NMR) spectra were recorded by a Bruker AV400 NMR spectrometer (made by Bruker, Karlsruhe, Germany) with a proton frequency at 200 MHz. Deuterated chloroform (CDCl_3_) was used as a solvent, and tetramethylsilane (TMS) was used as an internal standard. The viscosity–temperature relationship was measured from 20 °C to 80 °C at a heating rate of 2 °C/min using a TA instrument (AR-2000 rheometer) with a 25 mm diameter. Differential scanning calorimetry (DSC) was conducted on the blends in hermetically sealed pans. The heating and cooling experiments were performed at 5, 10, 15, and 20 °C/min with Perkin-Elmer DSC7, made in Waltham, MA, USA. The sample (10 mg) was sealed under nitrogen in aluminum pans. Temperature ramping DSC during curing was performed from 50 to 350 °C. Thermal stability of samples was measured with a Mettler SDTA 851 thermogravimetric analyzer (TGA, made in Zurich, Switzerland) at a heating rate of 10 °C/min. The tensile and flexural strengths were measured by an RGM-4100 electronic material testing system, made in Shenzhen, China. 

## 3. Results and Discussion

### 3.1. Synthesis of PHS, AB and ASB

**Figure 1:**
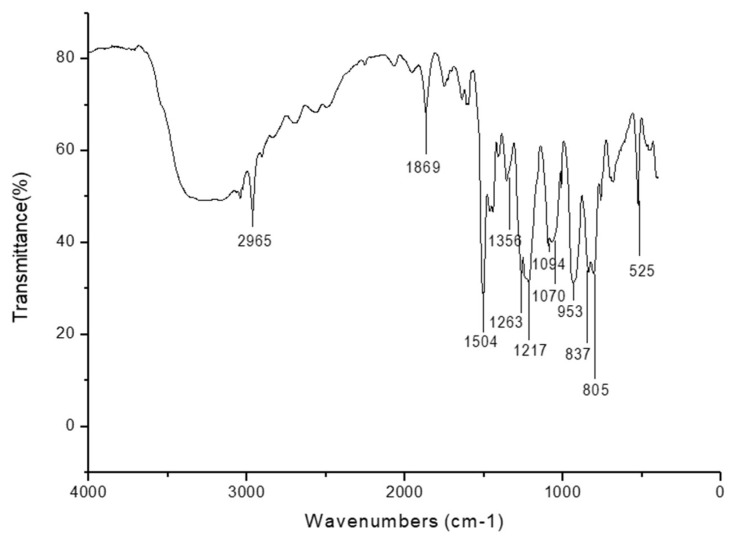
The FTIR spectrum of PHS.

In this work, the PHS compound was synthesized as a precursor for the preparation of high-performance acetylene-functional/silicon-containing benzoxazine. The chemical structure of PHS was characterized with FTIR as shown in Figure 1. In the FTIR spectrum of PHS, the absorption peaks at 1094 cm^−1^ of Si-O and 1263 cm^−1^ of Si-C indicated the reaction between Ar-OH and Si-Cl. The absorption peak at 2965 cm^−1^ was due to the stretching vibration of -CH_3_. The absorptions at 1869 cm^−1^ and 1782 cm^−1^ were due to the out-of-plane bending vibration of C-H of the symmetric substituted benzene ring, meanwhile the absorption at 806 cm^−1^ was the in-plane deformation vibration of C-H of the symmetric substituted benzene ring. According to the above results, PHS was synthesized successfully by hydroquinone and dimethyldichlorosilance.

**Figure 2:**
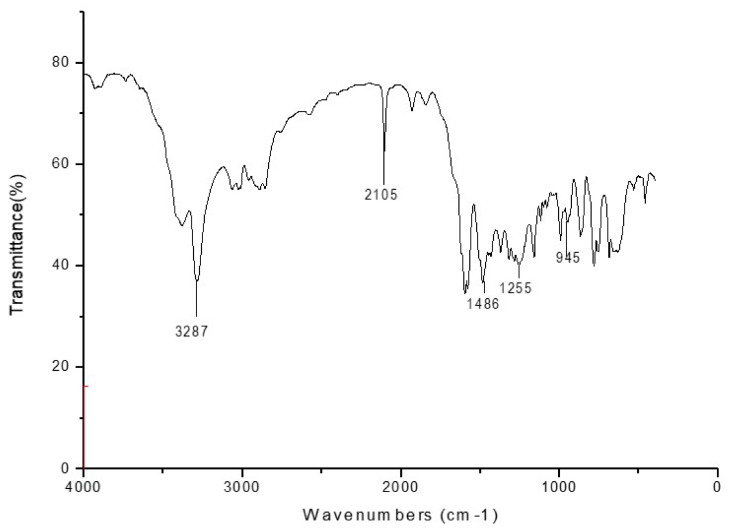
The FTIR spectrum of ASB.

The chemical structure of ASB was characterized with FTIR as shown in Figure 2. In the FTIR spectrum of ASB, the absorption peaks at 3287 cm^−1^ and 2105 cm^−1^ characterized the C≡C and C-H of the alkynyl group, respectively. The absorption peak at 1602 cm^−1^ characterized the benzene ring structure. The absorption peaks at 1486 cm^−1^ of -CH_2_, 1255 cm^−1^ of the tertiary amine and 1190 cm^−1^ of Ar-O characterized the oxazine ring. According to the above results, ASB was synthesized successfully by PHS, M-aminophenyl acetylene and formaldehyde.

**Figure 3:**
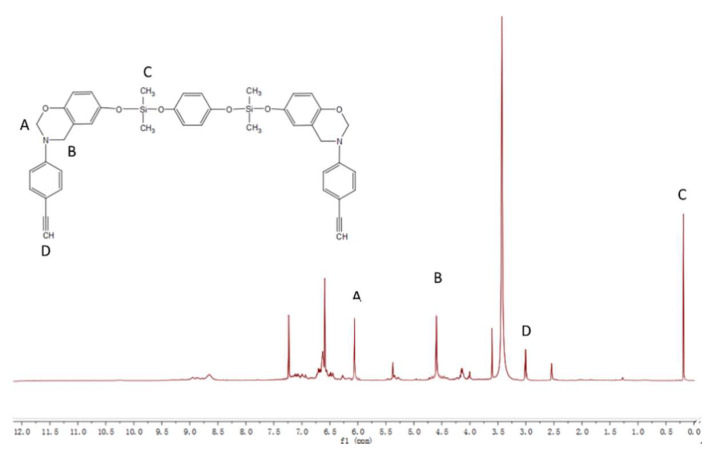
The ^1^H NMR spectrum of ASB.

^1^H NMR analysis was carried out to further confirm the molecular structure of the ASB monomer. The ^1^H NMR spectrum of ASB is shown in Figure 3. The proton resonances of N-CH_2_-O, Ar-CH_2_-N, Si-CH_3_ and ≡C-H appeared at 6.08 ppm, 4.58 ppm, 0.18 ppm and 3.05 ppm, respectively. The series of resonances at 6.62–7.30 ppm belonged to Ar-H protons. The formation of N-CH_2_-O and Ar-CH2-N indicated the successful synthesis of the oxazine ring, and further indicated the successful preparation of ASB. 

**Figure 4:**
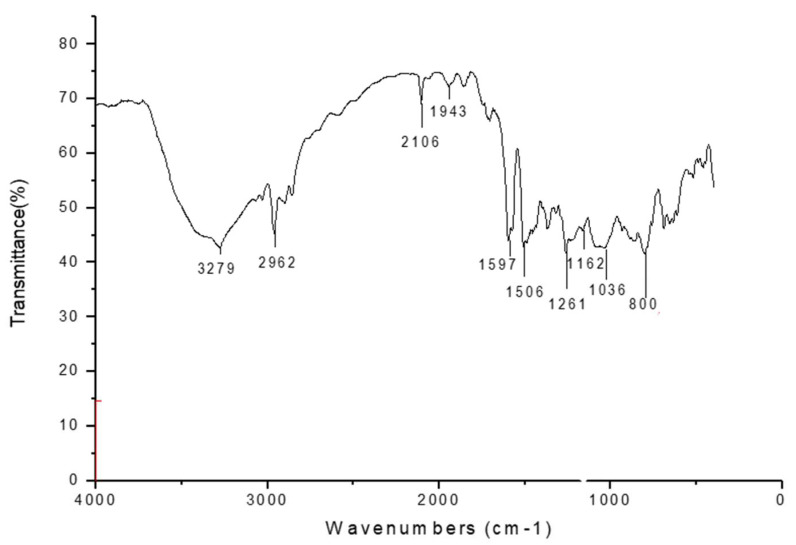
The FTIR spectrum of AB.

The chemical structure of AB was characterized with FTIR as shown in Figure 4. In the FTIR spectrum of AB, the absorption peaks at 3279 cm^−1^ and 2106 cm^−1^ characterized the C≡C and C-H of the alkynyl group, respectively. The absorption peak at 159 cm^−1^ characterized the benzene ring skeleton. The absorption peaks at 1506 cm^−1^ of -CH_2_- and 1261 cm^−1^ of the tertiary amine characterized the oxazine ring. According to the above results, AB was synthesized successfully by phenol, M-aminophenyl acetylene and formaldehyde.

### 3.2. Rheological Analysis

**Figure 5:**
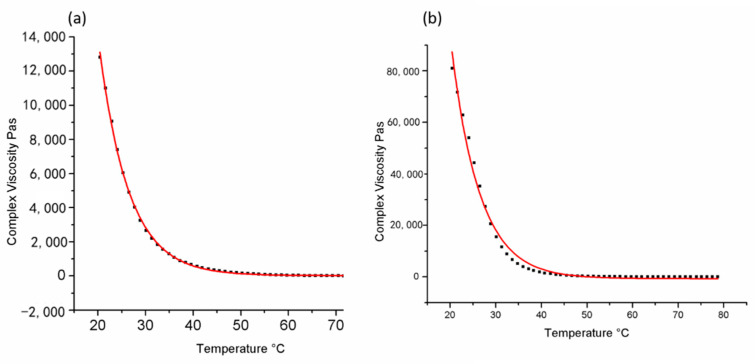
The viscosity–temperature relation of AB (**a**) and ASB (**b**).

The viscosity–temperature relations of AB and ASB were measured at a heating rate of 2 °C/min. As shown in Figure 5, the viscosity of both resins decreased dramatically with temperature increases from 20 °C to 40 °C, decreased gently with temperature increases from 40 °C to 60 °C, and finally became steady with temperature increases from 60 °C to 80 °C. Compared with AB, the ASB had an extremely high viscosity above 80,000 Pa·S at 20 °C. Nevertheless, when the temperature was above 60 °C, the viscosity of ASB was about 5.0 Pa·S, which was approximate to AB (4.5 Pa·S). Therefore, the results showed that the ASB resin had as wide a processing window as AB.

### 3.3. DSC Analysis

**Figure 6:**
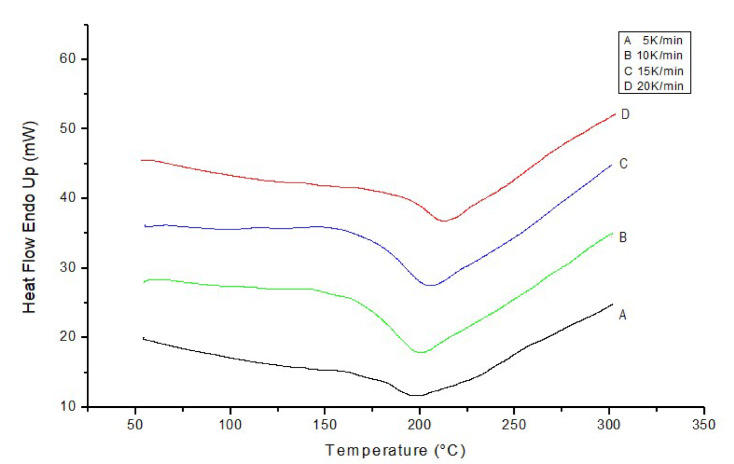
Curing exothermic curve under different heating rates of AB.

AB and ASB resins could both be thermally cured by polymerization of the acetylene-functional group and oxazine ring [11]. Figure 6 shows the DSC curing exothermic curve of AB at the heating rates of 5 °C/min, 10 °C/min, 15 °C/min and 20 °C/min, respectively. The exotherm was attributed to the cross-linking reaction. The DSC curves of AB showed wide exothermic peaks, which meant that the exothermic peaks of the oxazine ring-opening polymerization overlapped with the acetylene-functional group. It can be seen that the characteristic temperatures also gradually drifted towards higher temperatures with higher heating rates due to the thermal hysteresis effect. The initial temperatures, peak temperatures and end temperatures of the curing exotherm at different heating rates are tabulated in Table 1. 

**Figure 7:**
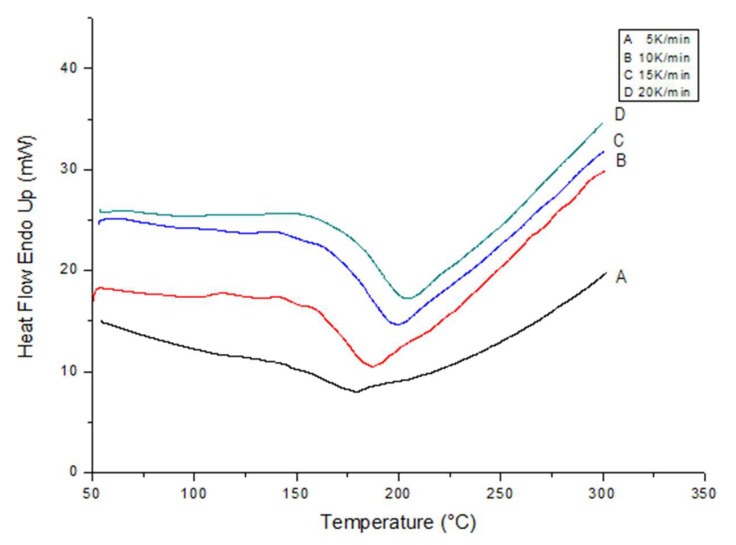
Curing exothermic curve under different heating rates of ASB.

Similarly, Figure 7 shows the DSC curing exothermic curve of ASB at the heating rates of 5 °C/min, 10 °C/min, 15 °C/min and 20 °C/min, respectively. The DSC curves of ASB also showed wide exothermic peaks, which meant that the exothermic peaks of the oxazine ring-opening polymerization overlapped with the polymerization of the acetylene-functional group, the same as AB. The initial temperatures, peak temperatures and end temperatures of the curing exotherm at different heating rates are tabulated in Table 2. As shown in both Table 1 and Table 2, the characteristic temperatures at the 5 °C/min heating rate were lowest compared with other heating rates. Obviously, the corresponding characteristic temperatures of ASB were lower than AB. Moreover, compared with AB, the initial curing temperature and peak temperature of ASB were, respectively, decreased from 169 °C to 146 °C and from 198 °C to 180 °C at the 5 °C/min heating rate. As we know, many research studies have proven that acetenyl had a catalytic effect on the ring-opening polymerization of benzoxazine [25]. The DSC results indicated that siloxane might also have a catalytic effect on the polymerization of either benzoxazine or acetenyl, or maybe both.

### 3.4. Research on Resin Curing Reaction Kinetics 

The reactions in the curing process of AB and ASB are complicated because of the ring-opening polymerization of the benzoxazine group, cyclization and crosslink of arylacetylene. The whole curing process has different groups to react, and even the same groups have diverse curing mechanisms. Curing kinetics equation analysis plays an important role in building the basis for a variety of quantitative and semi-quantitative research on the curing system and calculation of the reaction kinetics parameters [19].

**Figure 8:**
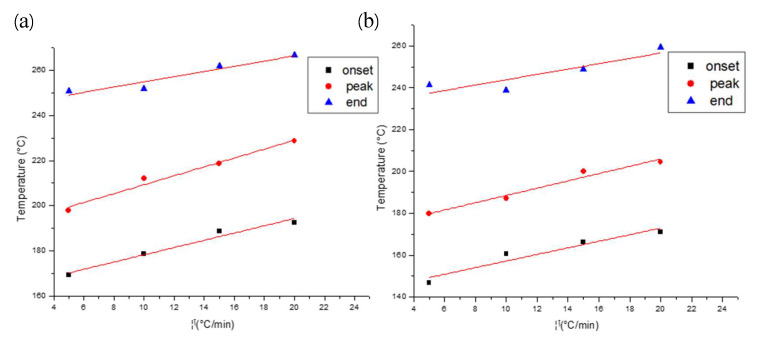
Relation between resin curing temperature and heating rate for AB (**a**) and ASB (**b**).

The pictures in Figure 8 show the relations between resin curing temperature and heating rate for AB (Figure 8a) and ASB (Figure 8b). According to the extrapolation of β = 0, it could be calculated that the initial temperature, peak temperature and end temperature of AB were, respectively, 162 °C, 189 °C and 243 °C. Similarly, the initial temperature, peak temperature and end temperature of ASB were 141 °C, 171 °C and 231 °C, respectively. This indicated that the introduction of PHS to acetylene-containing benzoxazine could effectively reduce the curing temperature by about 20 °C, which is beneficial to industrial production for its economic benefits.

The Kissinger method is commonly used to deal with DSC curve data for all parameters of the curing reaction kinetics. According to the Kissinger method, it is assumed that the curing reaction rate maximizes at the place of peak temperature in the DSC curve. Further transformed, the relationship between the reaction activation energy and temperature can be obtained as illustrated in Equation (1).

(1) $$\frac{d\left[\ln\left(\beta /T_{p}^{2}\right)\right]}{d\left(1/T_{p}\right)}=-\frac{E}{R}$$

where *β* is heating rate (K/min); *T*_p_ is curing peak temperature (K); E is activation energy (kJ/mol); R is the ideal gas constant (8.314 J/mol·K).

**Figure 9:**
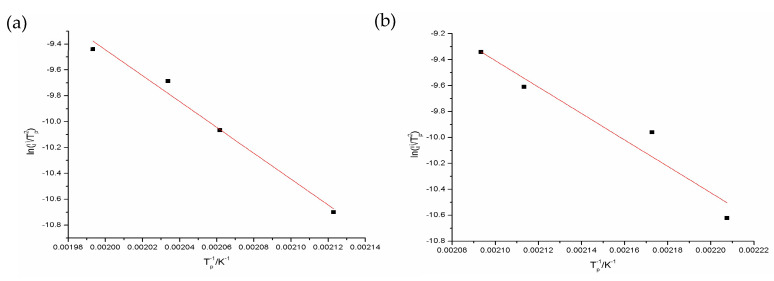
Kissinger method fitting figure of (**a**) AB and (**b**) ASB.

The apparent activation energy of the AB curing reaction could be calculated to obtain a value of 84.7 kJ/mol according to the slope of the curve in Figure 9a, and ASB obtains a value of 83.1 kJ/mol according to Figure 9b. The results showed the apparent activation energy of ASB was slightly lower than AB. Therefore, it could be indicated that ASB resins possessed better cure processability than the BA monomer, which was consistent with the results of DSC.

### 3.5. TGA Analysis

**Figure 10:**
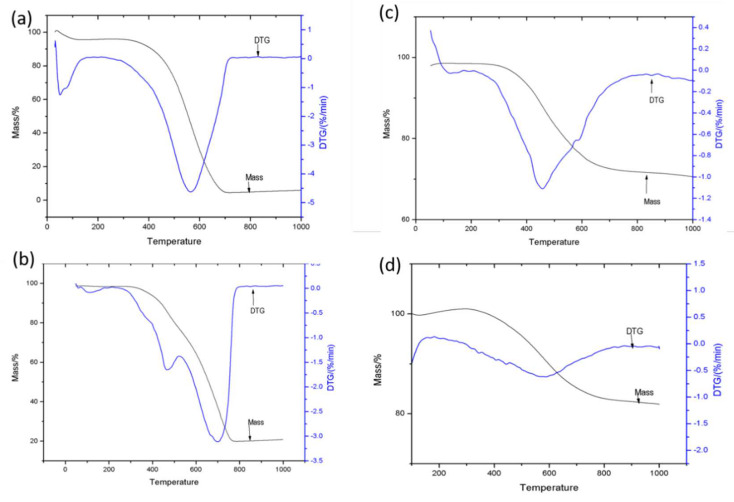
TGA curves in air: (**a**) AB and (**b**) ASB; TGA curves in nitrogen: (**c**) AB and (**d**) ASB.

Thermogravimetric analysis (TGA) in air and nitrogen was conducted to provide the thermal stability information of cured AB and ASB resins. The TGA curves of two kinds of benzoxazine resin in air and nitrogen atmosphere are shown in Figure 10 below. The temperature of a weight loss of 5% (T_d5_) and the char yield (Y_c_) at 1000 °C are tabulated in Table 3.

As shown in Table 3, all the T_d5_ results were above 360 °C, and the Y_c_ results at 1000 °C of both resins in nitrogen were above 72%. This indicated that the acetylene-functional benzoxazine possessed good thermal stability because the acetylene group could form a highly cross-linked network structure in order to stabilize the Mannich Bridge. The TGA results in air showed that the T_d5_ of ASB was 20 °C higher than AB, and the Y_c_ at 1000 °C of ASB was 19% more than AB. The results in nitrogen showed that the T_d5_ of ASB was about 50 °C higher than AB and the Y_c_ at 1000 °C of ASB was about 8% more than AB. It was supposed that a cross-linked benzoxazine containing silicon was formed to give a thermally stable structure when heated to a high temperature, which has been proven earlier [17]. 

The residual weight of ASB was more than 20% (as shown in Figure 10c) at a temperature of 1000 °C in the air atmosphere, because of its main chain containing silicon element. There is a possibility of the production of silicon compounds at high temperature and in an oxygen-containing atmosphere. The benzoxazine resin without silicon was just almost 2% in the air atmosphere, because it only contained C, H, O and N elements and those elements escape in the form of gaseous oxide at high temperatures. While the benzoxazine resin containing silicon residue weighed more than 20%, it might be that the silicon element generated stable silicon oxide on the surface of samples at high temperature and in an oxygen-containing atmosphere. 

### 3.6. Mechanical Property Analysis

The introduction of ether linkage into the main chain of the molecule has always been a good way to improve the brittleness of materials. The mechanical properties of AB and ASB were measured to identify the influence of ether linkage on ASB. As shown in Table 4, the results showed that the impact strength of ASB was higher than AB by 35.4%, which was consistent with our expectation. These distinctive improvements of ASB in the impact strength could be attributed to a flexible ether linkage group in the main chain. Interestingly, the results also showed that the tensile and flexural strengths of ASB were slightly higher than AB. As we know, the introduction of PHS led to a lower cross-linking density, which would decrease the tensile and flexural strength. This might be a result of an intermolecular Ar-O-Si-O-Ar group that was generated from the reaction between -Si-CH_3_ and the phenolic hydroxyl group derived from the ring-opening polymerization of benzoxazine [25].

## 4. Conclusions

In the current study, a high-performance acetylene-functional/silicon-containing benzoxazine monomer was successfully synthesized. FTIR and ^1^H NRM analyses confirmed the products. The viscosity of ASB was about 5 Pa·S when the temperature was above 60 °C, which showed that the ASB resin had a wide processing window. The DSC analysis concluded that introduction of PHS into AB had decreased the initial curing temperature and peak temperature by around 20 °C, respectively, which is beneficial to industrial production for economic benefits. The apparent activation energy of the ASB curing reaction was 83.1 kJ/mol, which was slightly lower than AB. The TGA results showed that ASB had a higher thermal stability than AB. The T_d5_ of ASB was 380 °C in nitrogen and 485 °C in air, while the Y_c_ at 1000 °C was 21% in air and 80% in nitrogen. It might be that stable silicon oxide was generated on the surface of samples at high temperature and in an oxygen-containing atmosphere. The mechanical property results showed that the impact strength of ASB was higher than AB by 35.4%, which was attributed to a flexible ether linkage group in the main chain. The results also showed that the tensile and flexural strengths of ASB were slightly higher than AB, which might be a result of the production of an intermolecular Ar-O-Si-O-Ar group generated from the reaction between the phenolic hydroxyl group and -Si-CH_3_.

## Figures and Tables

**Table 1:** The characteristic temperatures at various heating rates of AB.

Heating Rate (°C/min)	Initial (°C)	Peak (°C)	End (°C)
5	169.2	197.9	250.8
10	178.6	211.9	251.7
15	188.8	218.6	261.7
20	192.5	228.6	266.7

**Table 2:** The characteristic temperatures at various heating rates of ASB.

Heating Rate (°C/min)	Initial (°C)	Peak (°C)	End (°C)
5	146.6	179.9	241.2
10	161.8	187.1	238.8
15	166.2	200.0	248.6
20	171.1	204.6	259.2

**Table 3:** TGA analysis results of AB and ASB in air and nitrogen.

Resin	In Air		In Nitrogen	
	T_d5_/°C	Y_c_ at 1000 °C	T_d5_/°C	Y_c_ at 1000 °C
AB	360	2%	436	72%
ASB	380	21%	485	80%

**Table 4:** Mechanical property results of AB and ASB.

Mechanical Property	AB	ASB
Tensile Strength/MPa	76.8 ± 2.85	78.2 ± 1.85
Flexural Strength/MPa	141.8 ± 5.87	149.3 ± 4.56
Impact Strength/kJ/m^2^	24.2 ± 2.17	32.8 ± 2.89
